# Doença Coronariana de Pequenos Vasos e Balões Revestidos com Medicamentos: Hora de Reconsiderar Nossa Estratégia?

**DOI:** 10.36660/abc.20250652

**Published:** 2025-11-27

**Authors:** Guy F. A. Prado, Henrique Barbosa Ribeiro

**Affiliations:** 1 Hospital Israelita Albert Einstein São Paulo SP Brasil Hospital Israelita Albert Einstein, São Paulo, SP – Brasil; 2 University of Rome La Sapienza Department of Clinical and Molecular Medicine Roma Itália University of Rome La Sapienza – Department of Clinical and Molecular Medicine, Roma – Itália; 3 Instituto do Coração do Hospital das Clínicas da Faculdade de Medicina da Universidade de São Paulo São Paulo SP Brasil Instituto do Coração do Hospital das Clínicas da Faculdade de Medicina da Universidade de São Paulo, São Paulo, SP – Brasil; 4 Hospital Sirio-Libânes São Paulo SP Brasil Hospital Sirio-Libânes, São Paulo, SP – Brasil

**Keywords:** Intervenção Coronária Percutânea, Stents Farmacológicos, Doença da Artéria Coronariana

A intervenção coronária percutânea (ICP) com stents farmacológicos (SF) transformou drasticamente a revascularização coronária, reduzindo a reestenose e a necessidade de intervenções repetidas.^1^ No entanto, apesar dos grandes avanços na bioengenharia de stents e nas técnicas de procedimento, a implantação permanente de plataformas metálicas e revestimentos de polímeros continua associada à falha de stents a longo prazo, particularmente em vasos de pequeno calibre, com uma incidência anual de trombose de stent de aproximadamente 1–2%.^2^ Os mecanismos subjacentes incluem hiperplasia neointimal, neoaterosclerose, fratura de stent e a incapacidade de atingir remodelamento positivo, todos resultantes do "enjaulamento" dos vasos com suportes metálicos.^3^ Nesse contexto, o uso de balões farmacológicos (DCBs) para o tratamento de lesões de novo — incorporando a estratégia de "não deixar nada para trás" — surgiu como uma alternativa atraente para superar as limitações inerentes ao implante de stents.^4^

Nos últimos anos, um número crescente de estudos destacou a relevância clínica da angioplastia DCB para lesões de novo.^5^ A prevenção da implantação permanente do dispositivo na parede do vaso é particularmente atraente.^6^ Por outro lado, um objetivo fundamental da ICP é obter ganho luminal agudo, minimizando dissecções e fluxo prejudicado — um aspecto em que os stents mantêm uma clara vantagem. Ainda assim, a ICP com SF em artérias de pequeno calibre tem sido consistentemente associada a maiores taxas de falha da lesão-alvo. Nesse contexto, a angioplastia com DCBs é atraente. Evidências recentes sugerem resultados comparáveis entre DCBs e SFs em doenças de pequenos vasos; no entanto, delineamentos de não inferioridade e amostras pequenas têm limitado esses achados.^7^

Nesta edição do periódico, Gobbo et al. apresentam o manuscrito intitulado "Balões Revestidos com Paclitaxel versus Stents Farmacológicos na Doença Arterial Coronariana de Pequenos Vasos: Revisão Sistemática e Metanálise".^8^ O estudo aborda se a angioplastia com balão revestido com paclitaxel (PCB) fornece resultados comparáveis aos SF em pequenos vasos, por meio de uma análise conjunta de 12 estudos, incluindo 17.441 pacientes com acompanhamento de longo prazo (média de 32,4 meses). A análise compreendeu cinco ensaios clínicos randomizados e três estudos observacionais para o desfecho primário, revascularização da lesão alvo (RLA; n = 830). Os autores relataram taxas baixas e comparáveis de RLA: 6,6% para DCB versus 5,3% para SF (HR: 1,24; IC 95% 0,82–1,85; p = 0,46). Para o desfecho secundário, não houve diferenças significativas na perda tardia do lúmen (diferença média –0,09 mm; IC 95% –0,41 a 0,23; p = 0,57).

Esses achados devem ser interpretados à luz de três considerações principais. Primeiro, a ausência de diferenças significativas confirma que a ICP em pequenos vasos coronários é segura e eficaz com qualquer uma das estratégias. Segundo, a maioria dos ensaios incluiu apenas pacientes que haviam sido submetidos à pré-dilatação bem-sucedida antes da administração do medicamento antiproliferativo. Como resultado, os pacientes que apresentaram dissecções com limitação de fluxo foram sistematicamente excluídos tanto da análise quanto dos desfechos relatados. Na maioria dos casos, esses pacientes necessitaram de implante de stent de resgate ou tratamento médico conservador e, portanto, não foram representados na população do estudo. Terceiro, permanece uma questão importante sobre se o PCB poderia, em última análise, demonstrar superioridade, visto que estudos anteriores relataram que o implante de SF em pequenos vasos está associado a quase o dobro da taxa de falha do vaso-alvo em comparação com vasos maiores.^9^ Nesta fase, a presente metanálise não pode estabelecer tal conclusão. No entanto, o estudo PICCOLETO II — abrangendo 232 pacientes e comparando PCB com stent liberador de everolimus — demonstrou perda de lúmen tardia significativamente menor com PCB, alcançando não inferioridade e superioridade (0,04 mm vs. 0,17 mm; p = 0,001 e p = 0,03, respectivamente). Embora sem poder estatístico para desfechos clínicos, o acompanhamento de três anos mostrou menor incidência de eventos cardíacos adversos maiores (MACE) no grupo PCB (10,8% vs. 20,8%; p = 0,046), reforçando o potencial benefício da estratégia de "não deixar nada para trás".^10,11^ Na presente metanálise, não foram encontradas diferenças para MACE (HR: 1,01; IC 95% 0,76-1,33; p = 0,24), consistente com outros estudos anteriores. No estudo BASKET-SMALL 2, após três anos, a estimativa de Kaplan-Meier para MACE foi de 15% em ambos os grupos (HR 0,99; IC 95% 0,68-1,45; p = 0,95).^12^

Assim, podemos concluir que o PCB demonstrou ser uma estratégia segura e eficaz, com resultados comparáveis aos do SF em desfechos angiográficos e clínicos, a curto e longo prazo, para o tratamento de pequenos vasos coronários. No entanto, ensaios clínicos prospectivos randomizados, projetados com superioridade, particularmente para desfechos clínicos primários, seriam desejáveis para determinar qual estratégia é verdadeiramente ideal neste cenário. Dadas as taxas de eventos muito baixas observadas, tais ensaios exigiriam milhares de pacientes; portanto, metanálises e revisões sistemáticas bem conduzidas provavelmente continuarão sendo a abordagem mais viável para atingir esse nível de evidência.

**Figura 1:**
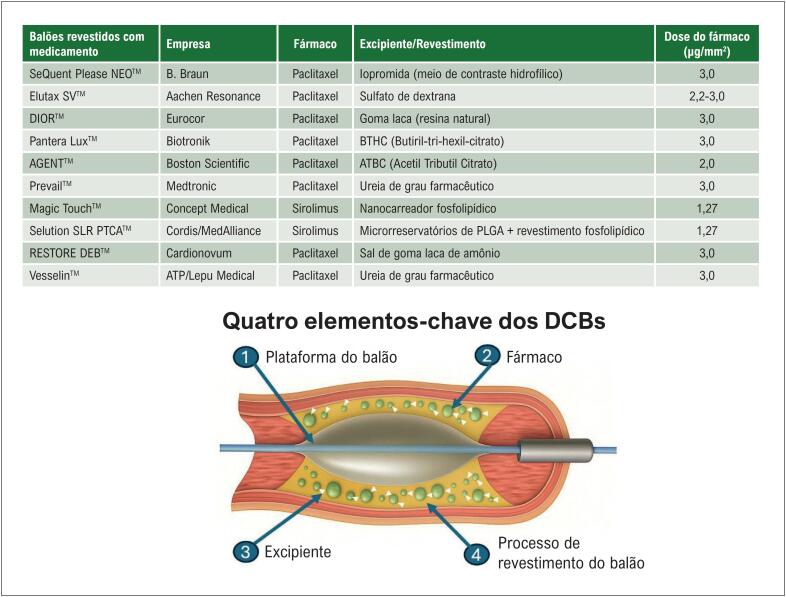
Balões farmacológicos coronários (DCBs) selecionados atualmente disponíveis e uma representação esquemática de seus principais componentes tecnológicos.

Outra questão crítica levantada neste estudo é se a angioplastia baseada em DCB pode ser considerada uma terapia de efeito de classe real, dada a ampla gama de dispositivos atualmente disponíveis no mercado.^13^ A resposta provável é não. As tecnologias de DCB diferem substancialmente entre as plataformas em relação à concentração do fármaco, ao revestimento do carreador do fármaco, ao tipo de agente antiproliferativo utilizado (paclitaxel versus família limus) e ao perfil de capacidade cruzada dos balões (Figura 1). Em conjunto, esses fatores tornam cada dispositivo único; portanto, cada DCB deve, pelo menos nesta fase, ser testado individualmente em ensaios clínicos adequadamente planejados. A metanálise atual de Gobbo et al.^8^ contribui com evidências valiosas para uma área de crescente relevância na cardiologia intervencionista. Ao mostrar resultados comparáveis entre PCB e SF em doenças de pequenos vasos, o estudo reforça a segurança e a eficácia da estratégia de DCB, ao mesmo tempo em que destaca questões sem resposta que ainda justificam investigações futuras. O desafio à frente é determinar se os DCBs podem não apenas igualar, mas eventualmente superar os SF em cenários clínicos e anatômicos selecionados, e se os refinamentos tecnológicos podem permitir que essa terapia se torne um novo padrão de tratamento na era de "não deixar nada para trás".
