# Supplementary material for: Doença Coronariana de Pequenos Vasos e Balões Revestidos com Medicamentos: Hora de Reconsiderar Nossa Estratégia?
Source: Arq Bras Cardiol. 2025 Nov 27;122(10):e20250652. [Article in Portuguese] doi: 10.36660/abc.20250652 (PMC12671718; doi:10.36660/abc.20250652)
Supplement: Translation

# Translation: Small-Vessel Coronary Disease and Drug-Coated Balloons: Time to Reconsider Our Strategy?

**DOI:** 10.36660/abc.20250652i

**Authors:** Guy F. A. Prado, Henrique Barbosa Ribeiro

**Affiliations:** 1 São Paulo SP Brazil Hospital Israelita Albert Einstein, São Paulo, SP – Brazil; 2 Rome Italy University of Rome La Sapienza – Department of Clinical and Molecular Medicine, Rome – Italy; 7 São Paulo SP Brazil Instituto do Coração do Hospital das Clínicas da Faculdade de Medicina da Universidade de São Paulo, São Paulo, SP – Brazil; 8 São Paulo SP Brazil Hospital Sirio-Libânes, São Paulo, SP – Brazil

**Keywords:** Percutaneous Coronary Intervention, Drug-Eluting Stents, Coronary Artery Disease

Percutaneous coronary intervention (PCI) with drug-eluting stents (DES) has dramatically transformed coronary revascularization, reducing restenosis and the need for repeat interventions.^1^ Nevertheless, despite major advances in stent bioengineering and procedural techniques, the permanent implantation of metallic platforms and polymer coatings remains associated with long-term stent failure, particularly in small coronary arteries, with an annual incidence of stent thrombosis of approximately 1–2%.^2^ The underlying mechanisms include neointimal hyperplasia, neoatherosclerosis, stent fracture, and the inability to achieve positive remodeling, all resulting from the "caging" of vessels with metallic struts.^3^ Against this background, the use of drug-coated balloons (DCBs) for the treatment of de novo lesions—embodying the "leave nothing behind" strategy—has emerged as an attractive alternative to overcome limitations inherent to stent implantation.^4^

In recent years, an increasing number of trials have underscored the clinical relevance of DCB angioplasty for de novo lesions.^5^ The avoidance of permanent device implantation into the vessel wall is particularly appealing.^6^ On the other hand, a key objective of PCI is to achieve acute luminal gain while minimizing dissections and impaired flow—an aspect where stents retain a clear advantage. Still, PCI with DES in small arteries has consistently been associated with higher rates of target lesion failure. In this context, a DCB-based angioplasty is compelling. Recent evidence has suggested comparable outcomes between DCBs and DES in small-vessel disease; however, non-inferiority designs and small sample sizes have limited these findings.^7^

In this edition of the journal, Gobbo et al. present the manuscript entitled "*Paclitaxel-Coated Balloons versus Drug-Eluting Stents in Small Vessel Coronary Artery Disease: A Systematic Review and Meta-Analysis*".^8^ The study addresses whether paclitaxel-coated balloon (PCB) angioplasty provides outcomes comparable to DES in small vessels, through a pooled analysis of 12 studies including 17,441 patients with long-term follow-up (mean 32.4 months). The analysis comprised five randomized controlled trials and three observational studies for the primary endpoint, target lesion revascularization (TLR; n = 830). The authors reported low and comparable rates of TLR: 6.6% for DCB versus 5.3% for DES (HR: 1.24; 95% CI 0.82–1.85; p = 0.46). For the secondary endpoint, there were no significant differences in late lumen loss (mean difference –0.09 mm; 95% CI –0.41 to 0.23; p = 0.57).

These findings should be interpreted in light of three key considerations. First, the absence of significant differences confirms that PCI in small coronary vessels is both safe and effective with either strategy. Second, most trials enrolled only patients who had undergone successful pre-dilation before the delivery of the antiproliferative drug. As a result, patients who experienced flow-limiting dissections were systematically excluded from both the analysis and the reported outcomes. In most cases, these patients required bailout stenting or conservative medical management and were therefore not represented in the study population. Third, an important question remains as to whether PCB could ultimately demonstrate superiority, given that previous studies have reported that DES implantation in small vessels is associated with nearly twice the rate of target vessel failure compared with larger vessels.^9^ At this stage, the present meta-analysis cannot establish such a conclusion. Nevertheless, the PICCOLETO II trial—encompassing 232 patients and comparing PCB with everolimus-eluting stent—demonstrated significantly less late lumen loss with PCB, achieving both non-inferiority and superiority (0.04 mm vs. 0.17 mm; p = 0.001 and p = 0.03, respectively). Although not powered for clinical endpoints, the three-year follow-up showed a lower incidence of major adverse cardiovascular events (MACE) in the PCB group (10.8% vs. 20.8%; p = 0.046), reinforcing the potential benefit of the "leave nothing behind" strategy.^10,11^ In the present meta-analysis, no differences were found for MACE (HR: 1.01; 95% CI 0.76–1.33; p = 0.24), consistent with other prior trials. In the BASKET-SMALL 2 trial, after three years, the Kaplan–Meier estimate of MACE was 15% in both groups (HR 0.99; 95% CI 0.68–1.45; p = 0.95).^12^

Thus, we may conclude that PCB has proven to be a safe and effective strategy, with outcomes comparable to DES in both angiographic and clinical endpoints, in the short and long term, for the treatment of small coronary vessels. Nevertheless, prospective randomized trials designed with superiority, particularly for primary clinical endpoints, would be desirable to determine which strategy is truly optimal in this scenario. Given the very low event rates observed, such trials would require thousands of patients; therefore, well-conducted meta-analyses and systematic reviews are likely to remain the most feasible approach to achieve this level of evidence.

Another critical issue raised in this study is whether DCB-based angioplasty can be regarded as an actual class-effect therapy, given the wide range of devices currently available on the market.^13^ The likely answer is no. DCB technologies differ substantially between platforms with respect to drug concentration, drug carrier coating, the type of antiproliferative agent used (paclitaxel versus limus family), and the crossability profile of the balloons (Figure 1). Altogether, these factors make each device unique; therefore, every DCB should, at least at this stage, be tested individually in adequately designed clinical trials. The current meta-analysis by Gobbo et al.^8^ contributes valuable evidence to an area of increasing relevance in interventional cardiology. By showing comparable outcomes between PCB and DES in small-vessel disease, the study reinforces the safety and efficacy of the DCB strategy while also highlighting unanswered questions that still warrant future investigation. The challenge ahead is to determine whether DCBs can not only match but eventually surpass DES in selected clinical and anatomical scenarios, and whether technological refinements may enable this therapy to become a new standard of care in the "leave nothing behind" era.

**Figure 1:** Selected currently available coronary drug-coated balloons (DCBs) and a schematic representation of their core technological components.
